# Supplementary material for: Chitosan Scaffolds from Crustacean and Fungal Sources: A Comparative Study for Bone-Tissue-Engineering Applications
Source: Bioengineering (Basel). 2024 Jul 16;11(7):720. doi: 10.3390/bioengineering11070720 (PMC11273506; doi:10.3390/bioengineering11070720)
Supplement: Supplementary file 1 [file bioengineering-11-00720-s001.zip › bioengineering-3039634-supplementary.pdf]

bioengineering

MDPI

Supplementary Figures

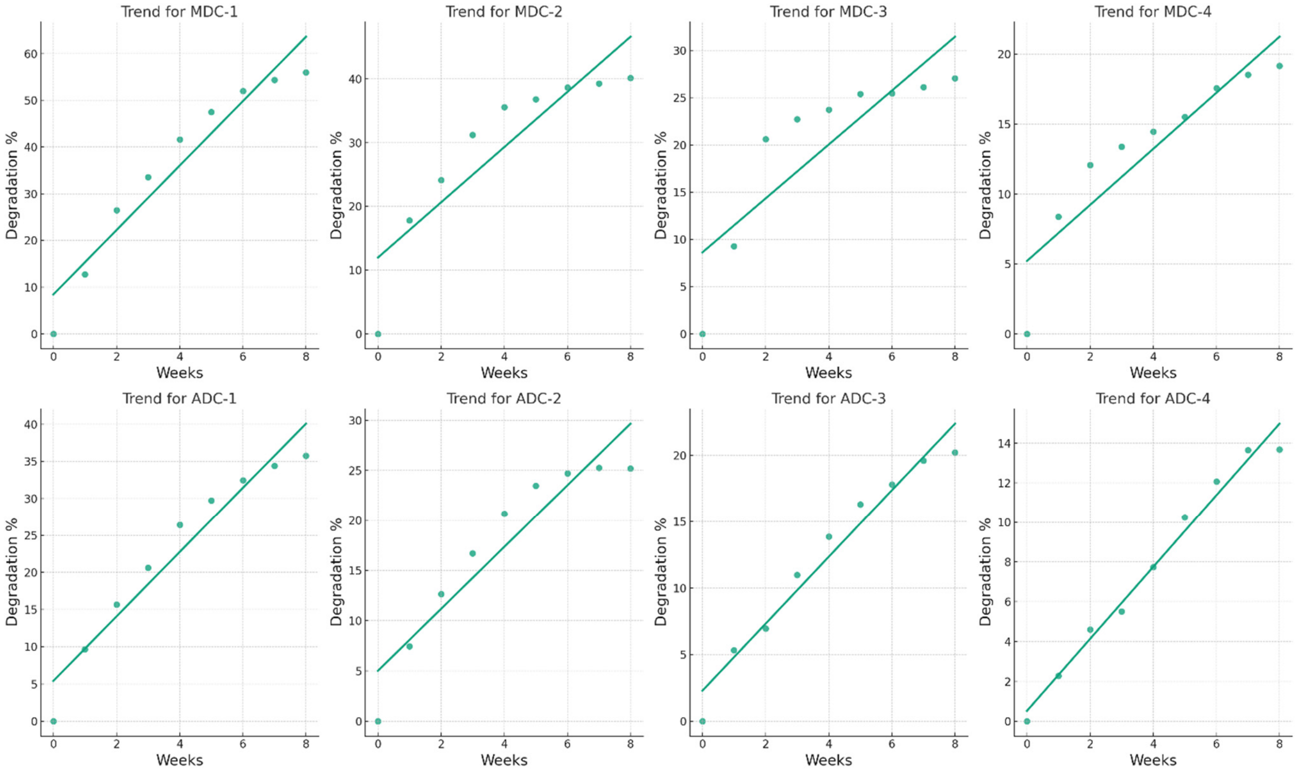

Figure S1. Linear regression for degradation percentage of MDC and ADC freeze-dried chitosan scaffolds containing 0, 10, 20 and 30(wt)% TCP minerals.

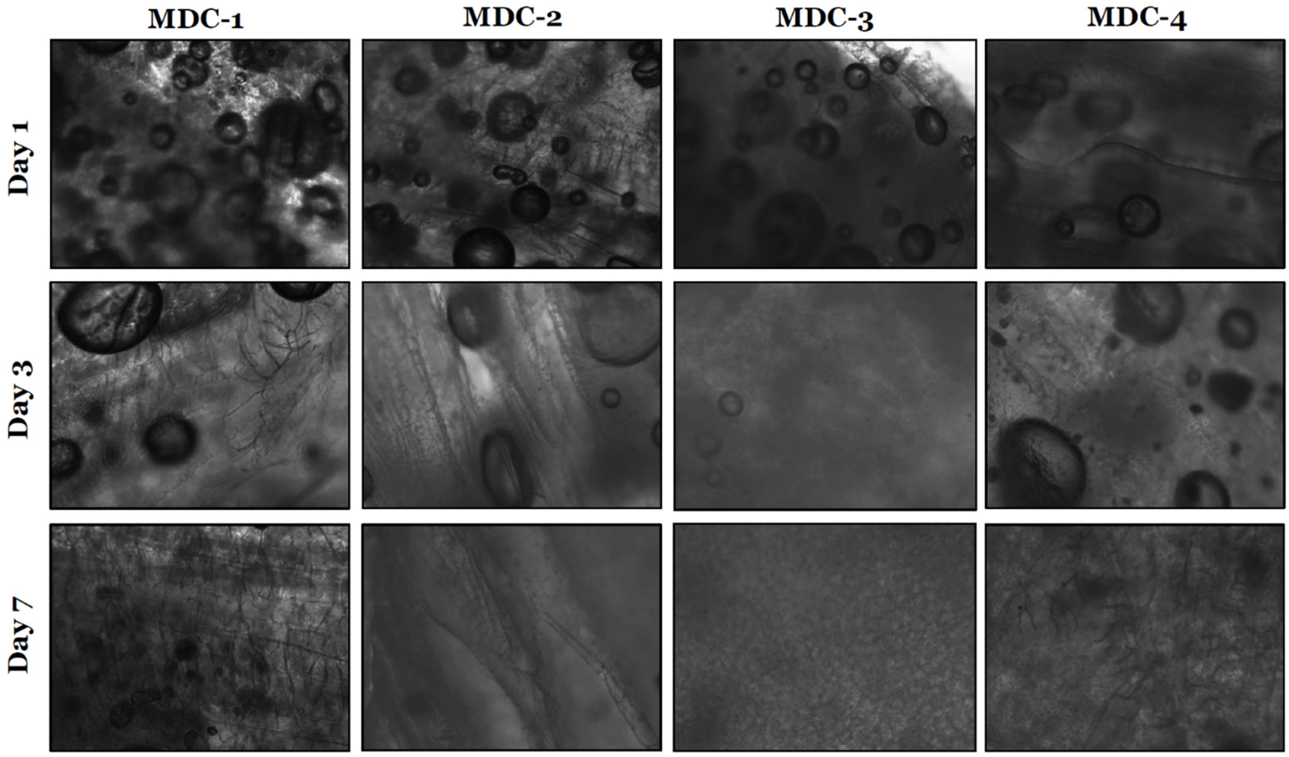

Figure S2. Sterility testing of MDC and ADC freeze-dried chitosan scaffolds containing 0, 10, 20 and 30(wt)% TCP minerals.

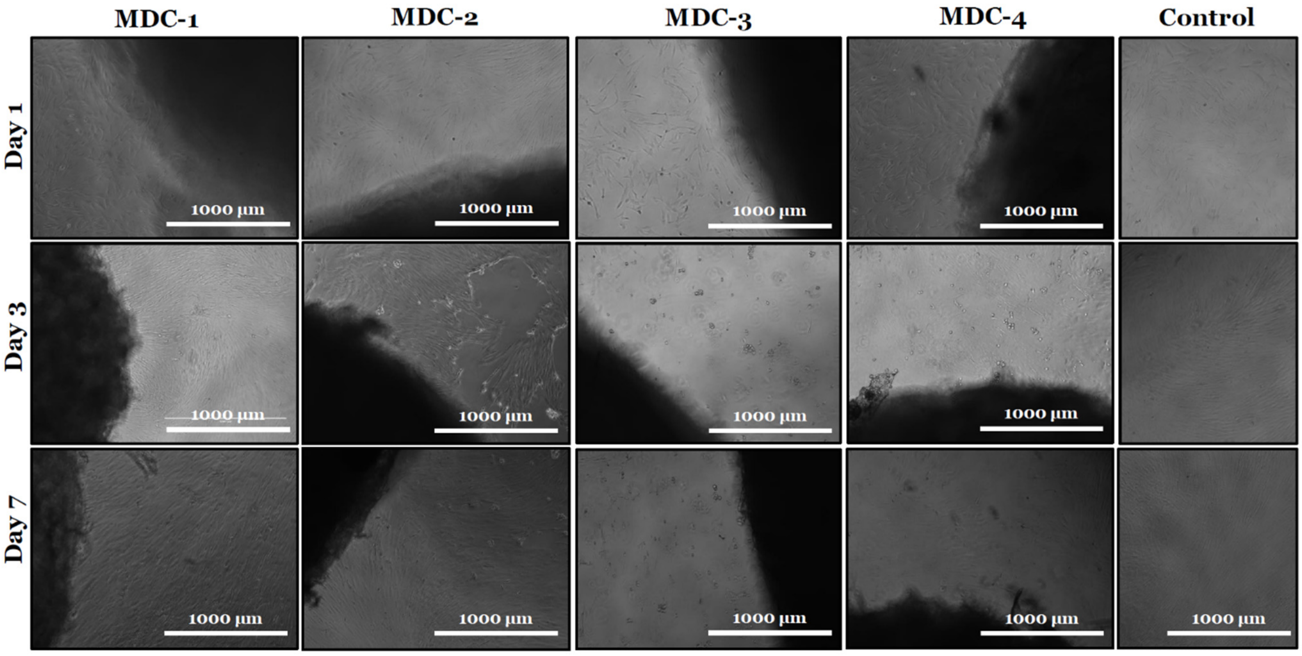

Figure S3. Direct toxicity assay for bone marrow mesenchymal stromal cells (BMMSCs) for fungal-derived (MDC) chitosan scaffolds doped with different concentrations of tricalcium phosphates minerals (0(wt)% (MDC-1), 10(wt)% (MDC-2), 20(wt)% (MDC-3) and 30(wt)% (MDC-4)). The cellular morphology of all scaffolds in comparison with the control group (absence of scaffold) is visualised at the junction of the cell-scaffold interface, using the EVOS microscope at a magnification of 4x.
